# Experimental Study on Differences in Clivus Chordoma Bone Invasion: An iTRAQ-Based Quantitative Proteomic Analysis

**DOI:** 10.1371/journal.pone.0119523

**Published:** 2015-03-20

**Authors:** Zhen Wu, Liang Wang, Zhengguang Guo, Ke Wang, Yang Zhang, Kaibing Tian, Junting Zhang, Wei Sun, Chunjiang Yu

**Affiliations:** 1 Dept Neurosurgery, Beijing Tiantan Hospital, Capital Medical University, China National Clinical Research Center for Neurological Diseases, Beijing, PR China; 2 Core Facility of Instruments, School of Basic Medicine, Chinese Academy of Medical Sciences, Institute of Basic Medical Sciences Peking Union Medical College, Beijing, PR China; 3 Dept Neurosurgery, Beijing Sanbo Brain Hospital, Capital Medical University, Beijing, PR China; Queen Mary Hospital, HONG KONG

## Abstract

Although a bone tumor, significant differences in the extent of bone invasion exist in skull base chordoma, which directly affect the extent of surgical resection, and have an impact on its prognosis. However, the underlying mechanism of the phenomenon is not clearly understood. Therefore, we used an iTRAQ-based quantitative proteomics strategy to identify potential molecular signatures, and to find predictive markers of discrepancy in bone invasion of clivus chordoma. According to bone invasive classification criteria, 35 specimens of clivus chordoma were calssified to be either endophytic type (Type I) or exophytic type (Type II). An initial screening of six specimens of endophytic type and six of exophytic was performed, and 250 differentially expressed proteins were identified. Through the GO and IPA analysis, we found evidence that the expression of inflammatory activity-associated proteins up-regulated in endophytic type, whereas the expression of cell motility-associated proteins up-regulated in exophytic ones. Moreover, TGFβ1 and mTOR signal pathway seemed to be related with bone invasion. Thus, TGFβ1, PI3K, Akt, mTOR, and PTEN were validated in the following 23 samples by immune histochemistry and Western blot. The expression levels of TGFβ1 and PTEN were significantly lower in the endophytic type than in the exophytic ones. It was found that TGFβ1 may play an important role in its bone invasion. The mechanisms may be related with conducting an increased inflammatory cell response and a decline in cytoskeletal protein expression. PTEN is confirmed to be associated with the degree of bone invasion. The PI3K/AKT/mTOR signaling pathway might be associated with the bone invasion, but still needs a larger sample size to be verified These results, for the first time, not only demonstrate the biological changes that occur in different growth patterns from the perspective of proteomics, but also provide novel markers that may help to reveal the mechanisms behind clivus chordomas.

## Introduction

Chordoma is a type of bone tumor originating from notochordal remnants. It often occurs in the body axis, including the skull base and sacral, and skull base chordoma accounts for about 32% of cases [1]. Radical surgery is the most effective treatment choice [2,3]. However, due to the depth of skull base chordoma and its proximity to complex structures, as well as tumor infiltration into adjacent bone, skull base chordoma resection is very difficult, and relapses after surgery are frequent occurrences. A retrospective study of our research group found that the skull base chordoma recurrence rates after 5 and 10 years are 52.9% and 88.3%, respectively [2].

The extent of skull base bone invasion in this kind of tumors is quite different. Bone invasion and destruction in some cases were quite heavy, which in some others were relatively light. Based on results of previous studies [2–4], as well as clinical practice, our research group discovered that the degree of bone invasion and the integrity of skull base dural barrier are independent risk factors affecting the clinical prognosis of skull base chordoma patients. In addition, Therefore, it is necessary to explore the causes and mechanisms of the differences in bone invasion.

The protein expression levels of Cadherins, Catenins, MMPs, Cathepsin B and uPA are related to the invasion of skull base chordoma [5,6], and these levels may affect treatment effect and prognosis. However, due to limitations of the experimental method, it is not yet possible to integrate and systematically analyze the proteins associated with chordoma bone invasion.

Integrated tumor proteomics research, especially differential proteomics and functional proteomics research, is a new tool of protein research [7]. Currently, there is only one report on chordoma proteomics research. The study analyzedthe differentproteins inchordomas and adjacent muscle tissues, but it failed to find specific protein associated with its prognosis[8]. Isobaric tags for relative and absolute quantitation (iTRAQ) is an isobaric labeling method used in quantitative proteomics by tandem mass spectrometry to determine the amount of proteins from different sources in a single experiment. It was a high-throughput quantification method which were more and more wildly used for quantitative proteomics.

This study grouped differences of clivus chordoma based on different bone infiltration imagings preoperatively, and used iTRAQ-based quantitative proteomic technology to analyze and compare the differentially expressed proteins in the corresponding subgroups. Furthermore, protein expression was confirmed by immune histochemical staining and Western blot.

## Materials and Methods

### 1. Case Selection criteria

The Institutional Review Board(IRB) of Beijing Tiantan Hospital,Capital Medical University approved the study. From January 2009 to January 2013, patients admitted to the Skull-base Ward, Neurosurgery Department of Beijing TianTan Hospital, Capital Medical University were included. All the patients were signed the Ethnic statements when they were enrolled. The documents were scaned and stored in the hospital. The written informed consent was obtained from the participants prior to their participation. In addition, the included patients should be primary untreated, and had lesions in the region of clivus, rather than in the foramen magnum, jugular foramen or spine. They all received tumor resection and were pathologically diagnosed as classical chordoma cases.

### 2. Bone invasive classification criteria

**Fig 1:**
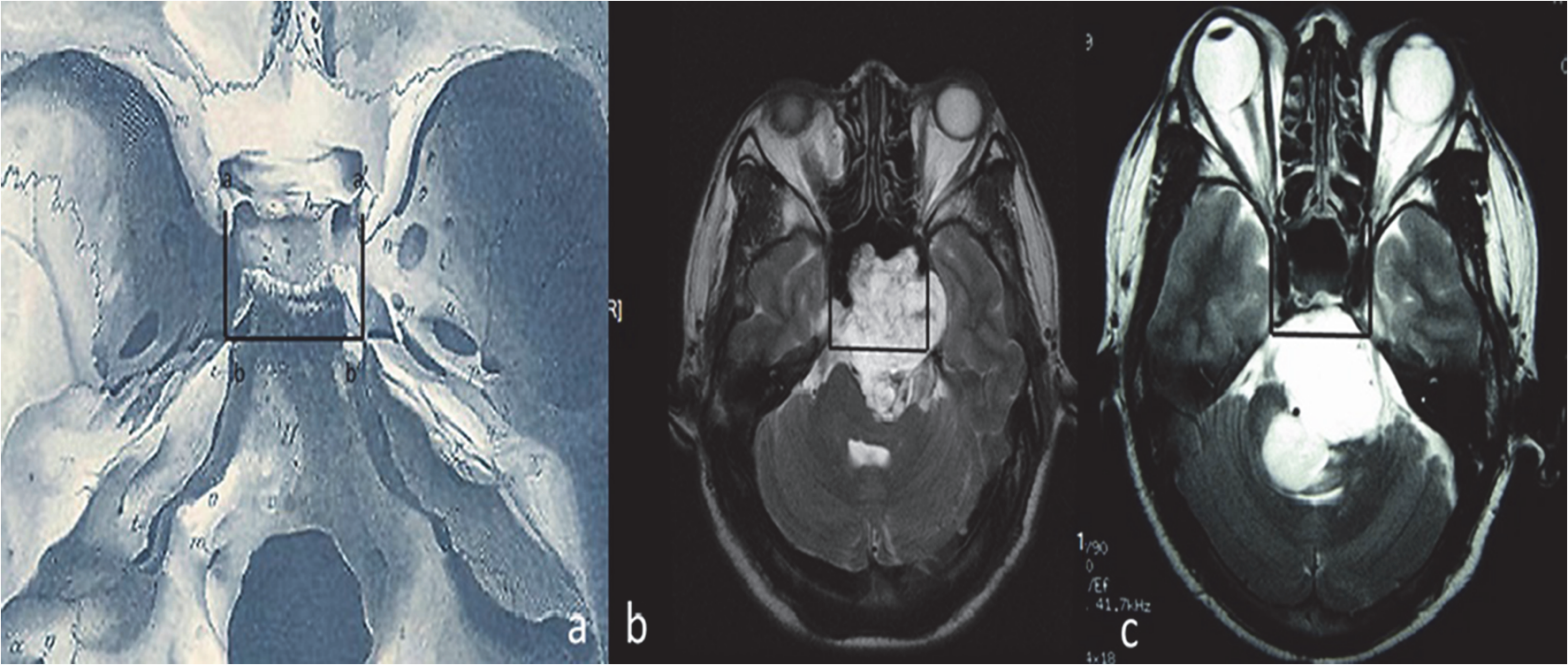
The Bone invasive classification criteria of clivus chordoma. Graph a shows the standard region in the baseline level of an anatomy image. Graph b shows a representative lesion of endophytic type (Type I); and Graph c shows a representative lesion of exophytic type (Type II).

Enrolledpatients were classified accordingto preoperative images(including plain and enhanced head MRI, thin layer skull base CT scanning and 3-D reconstruction). The maximum diameter at eyeball level of the T2 axial MR images was set as the baseline level, and the area of the bilateral carotid cavernous lateral walls connected to the bilateral petrous apex at the baseline level was set as the standard region. If at the baseline level, 50% or more of the tumor, which can invade the bone through all directions, was located within the standard region, and the clivus bone transformed like a “bubble” or a “dumbbell”, this kind of lesions was termed as endophytic type (Type I). If, on the baseline level, 50% or more of the tumor was located outside of the standard region, which had limited bone invasiveness, they may show “bulge-like” image from the clivus into the intracranial areas on the MR and CT scans, and this subgroup of tumors was termed exophytic type (TypeII) (see Fig. 1A-C). The selected cases were classified according to these criteria.

### 3. Specimen collection

Fresh tumor specimens were surgically resected immediately, divided into blocks, stored in liquid nitrogen, and fixed in 10% neutral formalin solution. They were paraffin fixed within one week and then stored in a 4° refrigerator for future use. All specimens underwent Hematoxylin and eosin (HE) staining before use to determine the percentage of tumor cells; specimens with fewer than 70% of the cells classified as tumor cells were excluded.

### 4. Proteomics methods

1. protein extraction and digestion: Eighty-milligram samples from each of the 12 frozen tissue samples selected for proteomics screening were rinsed with PBS, and each sample was then mixed with lysis buffer (50 mM Tris-HCl, 2.5 M thiourea, 8 M urea, 4% CHAPS, 65 mM DTT) to extract total protein. Cell debris was removed by centrifugation at 20,000 g for 45 min at 4°C. The total protein concentration of each sample was determined using the Bio-Rad RC DC Protein Assay. The proteins from each sample were pooled equally according to the total amount of protein and digested by filter-aided sample preparation combined with a microwave-assisted protein preparation method as previously described[9,10]. After digestion, peptides from the Type I and Type II samples were desalted on C18 columns (3 cc, 60 mg, Oasis) according to the manufacturer’s instructions, washed seven times with 500 μL 0.1% formic acid and eluted with 500 μL 100% ACN. Elutions were dried by vacuum centrifugation and stored at −80°C.
2. iTRAQ labeling: The digested chordoma samples were mixed at the same amountas internal standard. The chordoma samples and internal standard were labeled by 116,117, and118 iTRAQ. Labeling was performed according to the manufacturer’s protocol (ABsciex). The chordomasamples were mixed into onesample at the same amount and lyophilized.
3. 2D-LC/MS/MS: The pooled mixture from labeled samples was first fractioned by high-pH RPLC column from Waters (4.6mm×250mm, C18, 3μm). The samples were loaded onto the column in buffer A2 (pH = 10). The eluted gradient was 5–90% buffer B2 (90%ACN; pH = 10, flow rate, 1mL/min) for 60 min. The eluted peptides were collected as a fraction per minute, and the 60 fractions were pooled into 20 samples. Each sample was analyzed by RP C18 self-packing capillary LC column (75μm×100mm, 3μm). The eluted gradient was 5–30% buffer B1 (0.1% formic acid, 99.9% ACN; flow rate, 0.5 μL/min) for 100 min. TripleTOF 5600 were used to analyze the sample. The MS data were acquired with high sensitivity mode using the following parameters: 30 data-dependent MS/MS scans per every full scan; full scans was acquired at resolution 40,000 and MS/MS scans at 20,000; 35% normalized collision energy, charge state screening (including precursors with +2 to +4 charge state) and dynamic exclusion (exclusion duration 15 s); MS/MS scan range was 100–1800 m/z and scan time was 100 ms.
4. Database search: The MS/MS spectra were respectively searched against the SwissProt human database from Uniprot website (http://www.uniprot.org) using Mascot software version 2.3.02 (Matrix Science, UK). Trypsin was chosen as cleavage specificity with a maximum number of allowed missed cleavages of two. Carbamidomethylation (C) and iTRAQ 4-plex label was set as a fixed modification. The searches were performed using a peptide and product ion toleranceof 0.05 Da. Scaffold was used to further filter the database search results by decoy database method. The following filter was used in this study, 1% false positive rate at protein level and each protein with 2 unique peptides. After filtering the results by above filter, the peptide abundances in different reporter ion channels of MS/MS scan were normalized. The protein abundance ratio was based on unique peptide results.
5. GO functional analysis: All differential proteins identified by two approaches were assigned their gene symbol via the Panther database (http://www.pantherdb.org/). Protein classification was performed based on their functional annotations using Gene Ontology (GO) for biological process, and molecular function. When more than one assignment was available, all of the functional annotations were considered in the results.
6. IPA network analysis: All differential proteins were used for pathway analysis.

For this purpose, the SwissProt accession numbers were inserted into the Ingenuity Pathway Analysis (IPA) software (Ingenuity Systems, Mountain View, CA). This software categorizes gene products based on the location of the protein within cellular components and suggests possible biochemical, biological and molecular functions. Furthermore, proteins were mapped to genetic networks available in the Ingenuity and other databases and ranked by score. These genetic networks describe functional relationships between gene products based on known interactions in literature. Through the IPA software, the newly formed networks were associated with known biological pathways.

### 5. Immunohistochemical methods and analysis

Paraffin-embedded tumor tissue sections were immunohistochemically stained by the streptavidin peroxidase conjugatedmethod (SP method). Antibodies tested on the IHC included: rabbit anti-actin and anti-TGFβ1 (Santa Cruz Biotechnology Inc., Santa Cruz, CA, USA), anti-AKT, anti-mTOR, anti-PI3K, and anti-PTEN (Cell Signaling Technology, Inc., Danvers, MA, USA). Two independent pathologists viewed the sections under a microscopeunder good tissue structure and clear background conditions; they were unaware of the clinical data and prognoses of the selected patients. Positive signals of translational growth factor β1 (TGFβ1), PI3K, Akt, mTOR, and PTEN are yellowish brown particles appearing in the cytoplasm and/or nuclei. Using the staining intensity and the percentage of positive cells, we developed the following criteria: 1) 0 points for no stain, 1 point for light yellow, 2 points for yellowish-brown, 3 points for dark brown; 2) percentage of positive cells: 0 points for (0%), 1 point for (< 20%), 2 points for (20 to 50%), 3 points for (> 50%). A total score of less than 2 was denoted as negative, 3–4 was denoted as weakly positive, and 5–6 was denoted as strongly positive.

### 6. Western Blot Analysis

WBs of the additional 23 samples were performed to validate the proteomic quantitation of four selected candidate proteins (PI3K, AKt, mTOR, and PTEN). Electrophoresis and immunoblotting was performed on the protein extracts using the standard protocol, using 20 μg of protein per sample. Antibodies tested on the immunoblots included: rabbit anti-actin (Santa Cruz Biotechnology Inc., Santa Cruz, CA, USA), anti-AKT, anti-mTOR, anti-PI3K, and anti-PTEN (Cell Signaling Technology, Inc., Danvers, MA, USA). Following hybridization with the secondary antibody, the blots were incubated with Immun-Star horseradish peroxidase luminal/enhancer (Bio-Rad) and exposed onto Kodak Biomax MR Film (Eastman Kodak Company, Rochester, NY, USA).

### 7. Statistical Methods

Immunohistochemistry, western blot and clinical data were analyzed using the SPSS11.5 software package for statistical analysis. The differences in expression of TGFβ1, PI3K, AKt, mTOR, and PTEN, tumor volume, texture and degree of adhesion with TGFβ1, PI3K, AKt, mTOR and PTEN were analyzed by a chi-square test, and *p* < 0.05 was considered different.

## Results

### 1. Bone invasiveness classification and clinical outcomes

**Fig 2:**
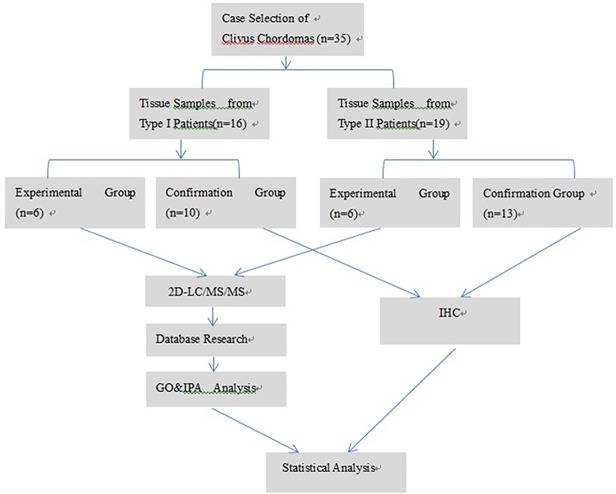
Workflow of the iTRAQ proteomic strategy. In this work, six pathologically verified tissue samples of endophyticclivus chordoma (Type I) and six samples of exophyticclivus chordoma (Type II)were digested with trypsin. The peptides were then sequencedusing 2D-LC/MS/MS and iTRAQ proteomic analysis. Subsequently, the peptide sequences were compared with the existing human database to acquire the protein list. The proteins were quantitatively analyzed usingPanther, and IPA was used to analyze biological functions. Several candidate proteins with interestingbiological functions were selected and further validated using IHC ofadditional 23 samples.Abbreviation: GO&IPA Analysis, Gene Ontology &Ingenuity Pathway Analysis; IHC,immunohistochemistry.

This study enrolled 35 patients, of which 12 were subjected to proteomic experiments (experimental group) and 23 were subjected to IHC and Western blot (confirmation group). Classifications were made in accordance with the aforementioned bone invasion criteria; there were six cases of Type I and II in the experimental group. There were 10 and 13 cases classified as Types I and II, respectively, in the confirmation group. There were no differences between the Type I and II patients in terms of sex, age, lesion size, tumor texture, or degree of adhesion in the validation group, as shown in Table 1. The workflow of the iTRAQ proteomic strategy is demonstrated in Fig. 2.

**Table 1:** The basic information of included patients.

Group	Type	Age(year)	Gender	Volume(ml)	Time for Chief complain (m)
1	I	16	1	68	12
1	I	46	2	58	12
1	I	15	1	36	3
1	I	32	1	36	24
1	I	44	1	10	4
1	I	58	2	55	48
1	II	21	1	50	3
1	II	47	1	6	6
1	II	36	1	45	2
1	II	18	1	24	24
1	II	17	1	40	1
1	II	60	2	25	7
2	I	40	2	11	1
2	I	40	2	21	6
2	I	28	1	14	9
2	I	50	1	36	12
2	I	30	1	16	6
2	I	13	1	18	0
2	I	46	2	40	24
2	I	48	2	30	6
2	I	23	2	12	5
2	I	51	1	24	3
2	I	44	2	5	1
2	I	42	1	12	12
2	I	22	1	14	4
2	II	50	2	168	12
2	II	39	2	14	3
2	II	12	2	60	6
2	II	47	1	60	7
2	II	56	1	15	24
2	II	15	1	70	1
2	II	25	2	25	36
2	II	57	1	11	3
2	II	16	2	80	12
2	II	38	1	27	24

Note: Group: 1, experimental group; 2, confirmation group. Type: I, endophytic type (type I); II, exophytic type (type II). Gender: 1, male; 2, female.

### 2. Identification of differentially expressed proteins in different growth pattern

This iTRAQ-labeling proteomic study compared the total proteomes of tissue from Type I patients (n = 6) with the proteomes of tissues from Type II patients (n = 6).Each individual sample in the two groups was separately analyzed. By querying the human IPI database with the Mascot algorithm, 2251 proteins were quantified.

Before performing comparative analysis between groups, the coefficient of variation was employed to filter out data with poor linearity among the biological replicates within each group. To maintain a low false-positive rate of comparative analysis between the groups, an average coefficient of variation of 0.2 (CV = 0.2) was accepted to filter out data with poor linearity.

Next, we applied a threshold of >1.5-fold and *p*<0.01 to identify proteins that were differentially expressed. A total of 250 proteins meeting the criteria were classified as differentially expressed. Among these proteins, 59 proteins were up-regulated and 191proteins were down-regulated in endophytic type (Type I) compared with exophytic type (Type II), as demonstrated in Table 2.

**Table 2:** The list of differentially expressed proteins.

Accession Number	Protein Names	Change Folds	Molecular Weight
P08729	Keratin, type II cytoskeletal 7	1.8	51 kDa
P35555	Fibrillin-1	0.6	312 kDa
P0CG38	POTE ankyrin domain family member I	0.6	121 kDa
P17661	Desmin	0.6	54 kDa
P51888	Prolargin	2	44 kDa
P16112	Aggrecan core protein	1.65	250 kDa
P21333	Filamin-A	0.6	281 kDa
Q15582	Transforming growth factor-beta-induced protein ig-h3	1.6	75 kDa
P02545	Prelamin-A/C	0.6	74 kDa
P24821	Tenascin	0.6	241 kDa
Q15063	Periostin	0.45	93 kDa
Q05707	Collagen alpha-1(XIV) chain	0.4	194 kDa
P13611	Versican core protein	0.4	373 kDa
P69905	Hemoglobin subunit alpha	0.65	15 kDa
Q12805	EGF-containing fibulin-like extracellular matrix protein 1	0.55	55 kDa
P07237	Protein disulfide-isomerase	1.8	57 kDa
P68371	Tubulin beta-4B chain	0.65	50 kDa
P21810	Biglycan	2.4	42 kDa
P10915	Hyaluronan and proteoglycan link protein 1	2.6	40 kDa
Q06828	Fibromodulin	1.95	43 kDa
P11047	Laminin subunit gamma-1	0.6	178 kDa
Q99879	Histone H2B type 1-M	0.6	14 kDa
Q8N257	Histone H2B type 3-B	0.55	14 kDa
P55268	Laminin subunit beta-2	0.6	196 kDa
Q9Y6C2	EMILIN-1	0.65	107 kDa
P02458	Collagen alpha-1(II) chain	1.6	142 kDa
Q9Y240	C-type lectin domain family 11 member A	2.75	36 kDa
P07451	Carbonic anhydrase 3	0.6	30 kDa
P02788	Lactotransferrin	1.7	78 kDa
Q7Z7G0	Target of Nesh-SH3	0.6	119 kDa
Q8N2S1	Latent-transforming growth factor beta-binding protein 4	0.4	173 kDa
P04179	Superoxide dismutase [Mn], mitochondrial	0.4	25 kDa
P12821	Angiotensin-converting enzyme	1.95	150 kDa
Q14314	Fibroleukin	1.75	50 kDa
Q16363	Laminin subunit alpha-4	0.6	203 kDa
O43852	Calumenin	1.55	37 kDa
P62979	Ubiquitin-40S ribosomal protein S27a	0.5	18 kDa
P50454	Serpin H1	0.6	46 kDa
P23142	Fibulin-1	0.4	77 kDa
Q7Z406	Myosin-14	1.6	228 kDa
Q8IUX7	Adipocyte enhancer-binding protein 1	1.55	131 kDa
P24844	Myosin regulatory light polypeptide 9	0.6	20 kDa
Q14112	Nidogen-2	0.6	151 kDa
P10412	Histone H1.4	0.6	22 kDa
P16403	Histone H1.2	0.65	21 kDa
Q8IVF2	Protein AHNAK2	1.95	617 kDa
P01876	Ig alpha-1 chain C region	1.65	38 kDa
P12429	Annexin A3	2.2	36 kDa
P02649	Apolipoprotein E	1.6	36 kDa
Q15113	Procollagen C-endopeptidase enhancer 1	0.5	48 kDa
P02461	Collagen alpha-1(III) chain	0.35	139 kDa
Q14573	Inositol 1,4,5-trisphosphate receptor type 3	1.55	304 kDa
P35442	Thrombospondin-2	0.6	130 kDa
P07942	Laminin subunit beta-1	0.65	198 kDa
P22626	Heterogeneous nuclear ribonucleoproteins A2/B1	0.55	37 kDa
Q05682	Caldesmon	0.6	93 kDa
P09936	Ubiquitin carboxyl-terminal hydrolase isozyme L1	2	25 kDa
P61978	Heterogeneous nuclear ribonucleoprotein K	0.6	51 kDa
O43491	Band 4.1-like protein 2	0.6	113 kDa
O60687	Sushi repeat-containing protein SRPX2	2.4	53 kDa
P14543	Nidogen-1	0.6	136 kDa
Q13740	CD166 antigen	1.9	65 kDa
P06702	Protein S100-A9	1.75	13 kDa
P98095	Fibulin-2	0.6	127 kDa
P06748	Nucleophosmin	0.45	33 kDa
P01911	HLA class II histocompatibility antigen, DRB1–15 beta chain	2.2	30 kDa
Q30154	HLA class II histocompatibility antigen, DR beta 5 chain	1.75	30 kDa
Q00839	Heterogeneous nuclear ribonucleoprotein U	0.55	91 kDa
P37837	Transaldolase	0.65	38 kDa
Q9BXN1	Asporin	0.2	43 kDa
P07339	Cathepsin D	1.55	45 kDa
O00339	Matrilin-2	0.45	107 kDa
P02511	Alpha-crystallin B chain	1.95	20 kDa
Q8WX93	Palladin	0.6	151 kDa
Q9UBX5	Fibulin-5	0.5	50 kDa
P39060	Collagen alpha-1(XVIII) chain	0.6	178 kDa
P07099	Epoxide hydrolase 1	1.9	53 kDa
P07737	Profilin-1	0.65	15 kDa
P20908	Collagen alpha-1(V) chain	0.55	184 kDa
Q9UKU9	Angiopoietin-related protein 2	1.8	57 kDa
O43405	Cochlin	3.35	59 kDa
Q31610	HLA class I histocompatibility antigen, B-81 alpha chain	1.6	40 kDa
P05109	Protein S100-A8	1.55	11 kDa
Q01995	Transgelin	0.3	23 kDa
P78539	Sushi repeat-containing protein SRPX	2.05	52 kDa
P31943	Heterogeneous nuclear ribonucleoprotein H	0.6	49 kDa
P55795	Heterogeneous nuclear ribonucleoprotein H2	0.5	49 kDa
O94832	Unconventional myosin-Id	0.6	116 kDa
P12107	Collagen alpha-1(XI) chain	1.55	181 kDa
Q14766	Latent-transforming growth factor beta-binding protein 1	0.5	187 kDa
P30043	Flavin reductase (NADPH)	0.65	22 kDa
P51812	Ribosomal protein S6 kinase alpha-3	0.65	84 kDa
P07910	Heterogeneous nuclear ribonucleoproteins C1/C2	0.6	34 kDa
Q9NR99	Matrix-remodeling-associated protein 5	0.45	312 kDa
Q14195	Dihydropyrimidinase-related protein 3	0.55	62 kDa
P37802	Transgelin-2	0.4	22 kDa
P38159	RNA-binding motif protein, X chromosome	0.65	42 kDa
P49747	Cartilage oligomeric matrix protein	0.4	83 kDa
P43243	Matrin-3	0.6	95 kDa
Q9BXJ4	Complement C1q tumor necrosis factor-related protein 3	2.65	27 kDa
Q13361	Microfibrillar-associated protein 5	0.4	20 kDa
O94769	Extracellular matrix protein 2	1.65	80 kDa
P14866	Heterogeneous nuclear ribonucleoprotein L	0.6	64 kDa
P01859	Ig gamma-2 chain C region	1.85	36 kDa
O75367	Core histone macro-H2A.1	0.65	40 kDa
Q13263	Transcription intermediary factor 1-beta	0.65	89 kDa
Q6UVY6	DBH-like monooxygenase protein 1	1.55	70 kDa
P26447	Protein S100-A4	0.45	12 kDa
P60981	Destrin	0.5	19 kDa
P13797	Plastin-3	0.5	71 kDa
Q07955	Serine/arginine-rich splicing factor 1	0.65	28 kDa
Q14192	Four and a half LIM domains protein 2	0.65	32 kDa
P01137	Transforming growth factor beta-1	0.65	44 kDa
P16070	CD44 antigen	0.6	82 kDa
P22352	Glutathione peroxidase 3	0.6	26 kDa
P46063	ATP-dependent DNA helicase Q1	0.6	73 kDa
Q9Y3Z3	SAM domain and HD domain-containing protein 1	0.65	72 kDa
P09429	High mobility group protein B1	0.55	25 kDa
O15232	Matrilin-3	0.45	53 kDa
P02763	Alpha-1-acid glycoprotein 1	1.65	24 kDa
Q02878	60S ribosomal protein L6	0.65	33 kDa
Q9GZM7	Tubulointerstitial nephritis antigen-like	0.6	52 kDa
Q14019	Coactosin-like protein	0.5	16 kDa
P21291	Cysteine and glycine-rich protein 1	0.65	21 kDa
P07305	Histone H1.0	0.65	21 kDa
Q9BXJ0	Complement C1q tumor necrosis factor-related protein 5	1.8	25 kDa
P62829	60S ribosomal protein L23	0.65	15 kDa
P62424	60S ribosomal protein L7a	0.65	30 kDa
O95865	N(G),N(G)-dimethylarginine dimethylaminohydrolase 2	0.5	30 kDa
P55060	Exportin-2	0.65	110 kDa
Q8WXF7	Atlastin-1	1.7	64 kDa
P39023	60S ribosomal protein L3	0.65	46 kDa
P16401	Histone H1.5	0.6	23 kDa
Q99969	Retinoic acid receptor responder protein 2	2.2	19 kDa
P84103	Serine/arginine-rich splicing factor 3	0.6	19 kDa
P12268	Inosine-5'-monophosphate dehydrogenase 2	0.65	56 kDa
P13861	cAMP-dependent protein kinase type II-alpha regulatory subunit	0.55	46 kDa
Q15165	Serum paraoxonase/arylesterase 2	1.65	39 kDa
Q16853	Membrane primary amine oxidase	0.6	85 kDa
Q6UX06	Olfactomedin-4	1.8	57 kDa
P31942	Heterogeneous nuclear ribonucleoprotein H3	0.6	37 kDa
P62136	Serine/threonine-protein phosphatase PP1-alpha catalytic subunit	0.65	38 kDa
Q12905	Interleukin enhancer-binding factor 2	0.65	43 kDa
Q9UKV3	Apoptotic chromatin condensation inducer in the nucleus	0.65	152 kDa
P35268	60S ribosomal protein L22	0.5	15 kDa
Q9UEY8	Gamma-adducin	0.6	79 kDa
O14979	Heterogeneous nuclear ribonucleoprotein D-like	0.55	46 kDa
P26599	Polypyrimidine tract-binding protein 1	0.55	57 kDa
Q92522	Histone H1x	0.65	22 kDa
P60866	40S ribosomal protein S20	0.65	13 kDa
Q07507	Dermatopontin	0.6	24 kDa
P51858	Hepatoma-derived growth factor	0.6	27 kDa
Q15417	Calponin-3	0.6	36 kDa
Q07092	Collagen alpha-1(XVI) chain	0.65	158 kDa
Q99983	Osteomodulin	0.4	49 kDa
P51991	Heterogeneous nuclear ribonucleoprotein A3	0.55	40 kDa
Q0ZGT2	Nexilin	0.65	81 kDa
Q53TN4	Cytochrome b reductase 1	0.6	32 kDa
P46776	60S ribosomal protein L27a	0.6	17 kDa
P17612	cAMP-dependent protein kinase catalytic subunit alpha	0.65	41 kDa
Q96D15	Reticulocalbin-3	0.6	37 kDa
Q99733	Nucleosome assembly protein 1-like 4	0.65	43 kDa
P61254	60S ribosomal protein L26	0.6	17 kDa
Q15818	Neuronal pentraxin-1	2.4	47 kDa
O43854	EGF-like repeat and discoidin I-like domain-containing protein 3	0.5	54 kDa
P52566	Rho GDP-dissociation inhibitor 2	0.55	23 kDa
P17302	Gap junction alpha-1 protein	1.6	43 kDa
P08138	Tumor necrosis factor receptor superfamily member 16	0.6	45 kDa
P35443	Thrombospondin-4	0.5	106 kDa
Q13185	Chromobox protein homolog 3	0.6	21 kDa
Q9UHB6	LIM domain and actin-binding protein 1	0.65	85 kDa
Q9BX66	Sorbin and SH3 domain-containing protein 1	0.65	143 kDa
P62266	40S ribosomal protein S23	0.65	16 kDa
Q16629	Serine/arginine-rich splicing factor 7	0.6	27 kDa
P08574	Cytochrome c1, heme protein, mitochondrial	0.6	35 kDa
P19013	Keratin, type II cytoskeletal 4	0.65	57 kDa
Q16576	Histone-binding protein RBBP7	0.55	48 kDa
P05186	Alkaline phosphatase, tissue-nonspecific isozyme	0.55	57 kDa
Q06033	Inter-alpha-trypsin inhibitor heavy chain H3	0.65	100 kDa
Q5JTB6	Placenta-specific protein 9	0.65	10 kDa
O75368	SH3 domain-binding glutamic acid-rich-like protein	0.65	13 kDa
P10620	Microsomal glutathione S-transferase 1	1.65	18 kDa
Q14956	Transmembrane glycoprotein NMB	2.15	64 kDa
P19652	Alpha-1-acid glycoprotein 2	1.8	24 kDa
O00231	26S proteasome non-ATPase regulatory subunit 11	0.65	47 kDa
P52597	Heterogeneous nuclear ribonucleoprotein F	0.5	46 kDa
Q13363	C-terminal-binding protein 1	0.65	48 kDa
Q75N90	Fibrillin-3	0.65	300 kDa
O60701	UDP-glucose 6-dehydrogenase	0.65	55 kDa
P50225	Sulfotransferase 1A1	0.65	34 kDa
O75821	Eukaryotic translation initiation factor 3 subunit G	0.6	36 kDa
Q9BUF5	Tubulin beta-6 chain	0.6	50 kDa
P55769	NHP2-like protein 1	0.6	14 kDa
Q92598	Heat shock protein 105 kDa	0.65	97 kDa
Q15717	ELAV-like protein 1	0.6	36 kDa
Q9Y6U3	Adseverin	0.4	80 kDa
Q08170	Serine/arginine-rich splicing factor 4	0.5	57 kDa
P63167	Dynein light chain 1, cytoplasmic	0.6	10 kDa
Q13595	Transformer-2 protein homolog alpha	0.6	33 kDa
P08294	Extracellular superoxide dismutase [Cu-Zn]	0.6	26 kDa
P67809	Nuclease-sensitive element-binding protein 1	0.65	36 kDa
Q68BL7	Olfactomedin-like protein 2A	1.6	73 kDa
Q8N163	DBIRD complex subunit KIAA1967	0.6	103 kDa
P04208	Ig lambda chain V-I region WAH	0.4	12 kDa
Q99439	Calponin-2	0.5	34 kDa
P61313	60S ribosomal protein L15	0.65	24 kDa
Q9H8L6	Multimerin-2	0.65	104 kDa
Q9UHX1	Poly(U)-binding-splicing factor PUF60	0.5	60 kDa
P51570	Galactokinase	0.65	42 kDa
P29762	Cellular retinoic acid-binding protein 1	0.4	16 kDa
P62241	40S ribosomal protein S8	0.6	24 kDa
P51911	Calponin-1	0.45	33 kDa
Q9HBL0	Tensin-1	0.6	186 kDa
P01861	Ig gamma-4 chain C region	2	36 kDa
Q53EL6	Programmed cell death protein 4	0.6	52 kDa
O43927	C-X-C motif chemokine 13	2.15	13 kDa
Q13247	Serine/arginine-rich splicing factor 6	0.55	40 kDa
P51674	Neuronal membrane glycoprotein M6-a	1.6	31 kDa
Q9Y625	Glypican-6	0.6	63 kDa
Q13243	Serine/arginine-rich splicing factor 5	0.55	31 kDa
Q9H4G4	Golgi-associated plant pathogenesis-related protein 1	0.55	17 kDa
Q9BRX8	Redox-regulatory protein FAM213A	1.7	26 kDa
Q92629	Delta-sarcoglycan	0.6	32 kDa
P56377	AP-1 complex subunit sigma-2	0.65	19 kDa
P17252	Protein kinase C alpha type	0.6	77 kDa
Q9BUT1	3-hydroxybutyrate dehydrogenase type 2	0.6	27 kDa
Q9UBQ7	Glyoxylate reductase/hydroxypyruvate reductase	0.65	36 kDa
O14980	Exportin-1	0.65	123 kDa
Q4V9L6	Transmembrane protein 119	0.6	29 kDa
Q6IBS0	Twinfilin-2	0.6	40 kDa
P62851	40S ribosomal protein S25	0.6	14 kDa
P24557	Thromboxane-A synthase	0.6	61 kDa
P47914	60S ribosomal protein L29	0.5	18 kDa
P55290	Cadherin-13	0.5	78 kDa
P04433	Ig kappa chain V-III region VG (Fragment)	1.6	13 kDa
P26583	High mobility group protein B2	0.6	24 kDa
O43809	Cleavage and polyadenylation specificity factor subunit 5	0.65	26 kDa
O95715	C-X-C motif chemokine 14	2.9	13 kDa
Q9UH65	Switch-associated protein 70	0.6	69 kDa
Q9NQ79	Cartilage acidic protein 1	0.6	71 kDa
P52943	Cysteine-rich protein 2	0.6	22 kDa
P30273	High affinity immunoglobulin epsilon receptor subunit gamma	0.65	10 kDa
Q15185	Prostaglandin E synthase 3	0.55	19 kDa
Q9BY50	Signal peptidase complex catalytic subunit SEC11C	0.55	22 kDa
Q8N3U4	Cohesin subunit SA-2	0.6	141 kDa

### 3. Interaction Networks and Functional Pathway Analysis

**Fig 3:**
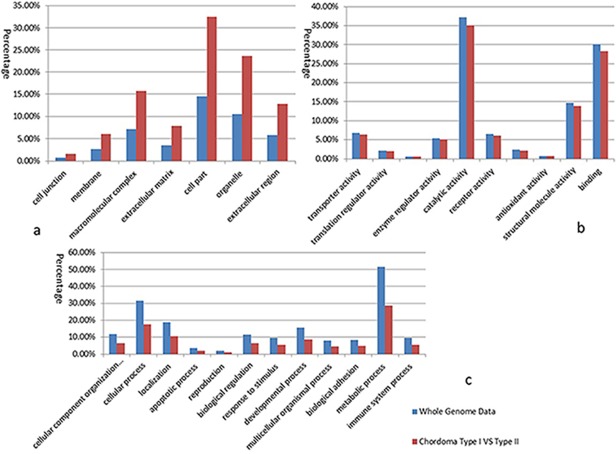
Panther analysis of endophyticclivus chordomas vs exophytic ones. Graph a shows cellular compartment analysis; Graph b shows molecular function analysis; and Graph c shows biological process analysis.

Functional pathway analysis was performed for the selected differentially expressed proteins to better understand their biological changes post-treatment. Panther analysis allowed us to elucidate the different functions and processes in which the 250 proteins are putatively involved compared with whole genome data. The cellular compartment, molecular function and biological process of the differentially expressed proteins are presented in Fig. 3. Organelles, extracellular complexes are significantly increased in chordomas, while metabolic process and cellular processes in biological processes significantly reduced, which may be related with the invasive growth of the tumor.

**Fig 4:**
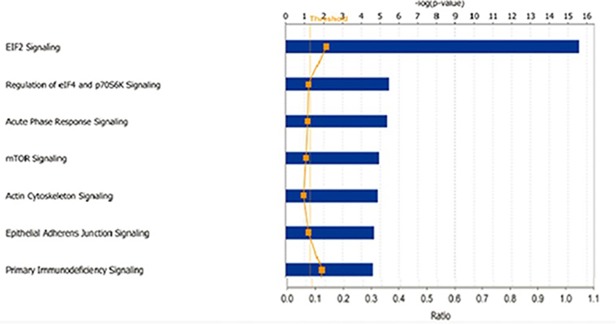
Mapping of the 250 proteins differentially expressed between endophytic clivus chordomas andexophyticones by IPA analysis. It illustrates the top 7 canonical pathways, while the mTOR pathway ranked the fourth.

Thereafter, we analyzed the 250 selected proteins using IPA and found that all of the proteins were eligible for network analysis (focus molecule) based on the IPA knowledgebase criteria. By employing the dataset of proteins that are differentially expressed between endophytic type (Type I) and exophytic type (TypeII), the role of these proteins in canonical pathways and disease &function were analyzed (Fig. 4).Among the top five canonical pathways returned, “EIF2 Signaling” and “Regulation of eIF4 and p70S6K signaling” were highly correlated with protein synthesis through the regulation of translation initiation, whereas “mTOR signaling” plays important roles in several cellular functions, particularly cell survival and proliferation.

According to the analysis, the main molecular functions of the differential proteins are primarily in the areas of cell motility, cell growth and proliferation, cellular organization and aggregation. The most important function of proteins is cell movement, and 91 types of proteins are related to cell movement. 34 proteins of these proteins are associated with tumor cell invasion, including VCAN, TGFB1, TGFβ1, FMOD and FLNA, which are common cell invasion-related proteins (Table 3).

**Table 3:** The list of molecules that are related to cellular movement.

Category	Diseases or Functions Annotation	p-Value	Molecules	Number of Molecules
Cellular Movement	cell movement	2.53E-16	ACAN,ALCAM,ANGPTL2,ANXA3,AOC3,APOE,ARHGDIB,BGN,CD44,CDH13,CLEC11A,CNN1,CNN2,COL18A1,COL2A1,COL3A1,COMP,CRYAB,CSE1L,CTBP1,CXCL13,CXCL14,DPT,DPYSL3,DSTN,EDIL3,EFEMP1,FBLN2,FBLN5,FBN1,FCER1G,FGL2,FHL2,FLNA,FMOD,GJA1,GLIPR2,GPM6A,HDGF,HMGB1,HMGB2,HNRNPA2B1,HNRNPK,HNRNPL,LAMB1,LAMC1,LIMA1,LMNA,LTF,MATN2,NEXN,NGFR,NPM1,NPTX1,OLFM4,ORM1,PALLD,PDCD4,PFN1,PON2,POSTN,PRKACA,PRKCA,RARRES2,RPS6KA3,S100A4,S100A8,S100A9,SOD2,SOD3,SRPX2,SWAP70,TAGLN2,TBXAS1,TGFB1,TGFBI,THBS2,THBS4,TNC,TNS1,UGDH,VCAN,YBX1	83
Cellular Movement	migration of cells	8.77E-13	ACAN,ALCAM,ANGPTL2,ANXA3,AOC3,APOE,ARHGDIB,BGN,CD44,CDH13,CLEC11A,CNN2,COL18A1,COL3A1,COMP,CRYAB,CSE1L,CTBP1,CXCL13,CXCL14,DPT,DPYSL3,EDIL3,FBLN2,FBLN5,FCER1G,FHL2,FLNA,FMOD,GJA1,GLIPR2,GPM6A,HDGF,HMGB1,HNRNPA2B1,HNRNPK,HNRNPL,LAMB1,LAMC1,LIMA1,LMNA,LTF,MATN2,NEXN,NGFR,NPM1,OLFM4,ORM1,PALLD,PDCD4,PFN1,PON2,POSTN,PRKACA,PRKCA,RARRES2,S100A4,S100A8,S100A9,SOD2,SOD3,SRPX2,SWAP70,TGFB1,TGFBI,THBS2,THBS4,TNC,TNS1,UGDH,VCAN	71
Cellular Movement	cell movement of tumor cell lines	1.70E-08	ARHGDIB,CD44,CDH13,CNN1,COL18A1,CSE1L,CTBP1,CXCL13,CXCL14,EFEMP1,FBLN2,FBLN5,FBN1,FHL2,FLNA,GJA1,HDGF,HNRNPA2B1,HNRNPK,PALLD,POSTN,PRKCA,RARRES2,RPS6KA3,S100A4,S100A8,SOD2,SRPX2,TAGLN2,TBXAS1,TGFB1,TGFBI,THBS2,TNC,YBX1	35
Cellular Movement	Invasion of cells	7.28E-08	ALCAM,CA3,CD44,COL18A1,CSE1L,CTBP1,CTSD,EFEMP1,FBLN1,FBLN2,FBLN5,FHL2,FLNA,FMOD,GJA1,HDGF,HMGB1,LAMC1,LTF,NPM1,PALLD,PDCD4,POSTN,PRKCA,PTGES3,S100A4,S100A9,SOD2,TAGLN,TAGLN2,TGFB1,TGFBI,THBS2,VCAN	34

Further classification analysis showed that the expression of inflammatory activity-associated proteins in endophytic type up-regulated, whereas the expression of cell motility-associated proteins in exophytictype up-regulated. In the endophytic chordoma tissues, inflammatory cells, especially phagocytic cells, had significantly increased motor function. For example, CXCL13, CXCL14 and CLEC11A promoted inflammatory cell movement; the expression of these molecules in the endophytic chordomas was significantly higher than that in exophyticones. In the exophytic chordoma tissues, the expression of tumor cell motility-associated proteins such as TGFβ1, HGDF, THBS2 and FBLN5 were significantly higher than that of the endophytic type. These proteins have a significant promotion effect on tumor migration.

**Fig 5:**
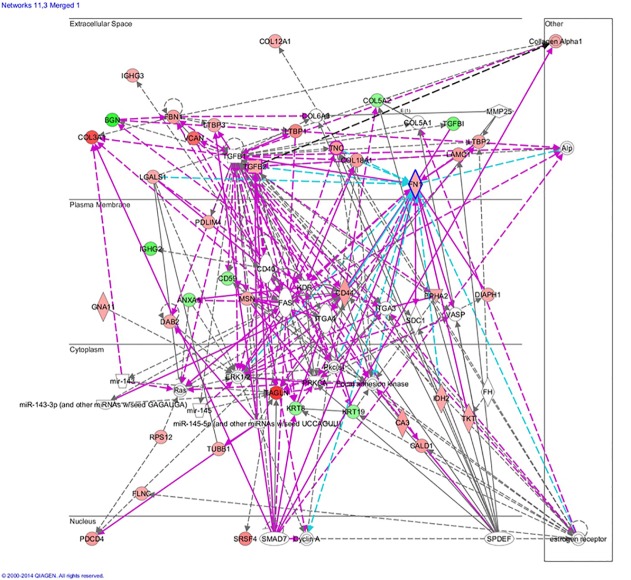
Protein synthesis and cellular function networks from IPA analysis. It shows the cellular function network and includes the functions “Cellular Movement, Cell Morphology, Connective Tissue, and Development and Function”, and “Cellular Movement, Cellular Development, Skeletal& Muscular System Development and Functio”. Proteins in red were up-regulated in endophytic clivus chordomas compared with exophytic ones, and proteins in green were down-regulated in endophytic clivus chordomas compared with exophytic ones.

From the IPA network analysis, twenty-one major overlapping interaction networks were identified, and the top six networks all had a score over twenty. By merging the “Cellular Movement, Cell Morphology, Connective Tissue Development and Function” (3rd network) and “Cellular Movement, Cellular Development, Skeletal and Muscular System Development and Function” (11th network), a molecular network were identified, as shown in Fig. 5. According to the score of the network and the result of the functional analysis, the most significantly related functions derived from these overlapping networks included protein metabolism and a series of cellular functions. The expression of many extracellular matrix proteins and cytoskeletal proteins, such as LAMA4, LAMB1, LAMB2, LAMBC1, NID1, NID2, NEXN, COTL1 and MYL9, changed significantly in endophytic chordomas, and TGFβ1,which was down-regulated, is the main protein upstream of these molecules. This molecular network shows that TGFβ1 can not only directly influence the migration of tumor cells, but also indirectly influence the movement of tumor cells by controlling the expression of cell matrix proteins and skeletal proteins. Based on these data, TGFβ1 may influence bone infiltration of clivus chordoma.

### 4. Validation of the Identified Differentially Expressed Proteins

Based on the result of the IPA analysis, 5 proteins (TGFβ1, PI3K, Akt, mTOR and PTEN) related to specific functions, such as proteins synthesis, cellular functionsand cancer, were selected for verification. Selection of proteins for validation was also performed on the basis of fold change of the proteins, the classification of proteins as secretory and the availability of antibodies. Validation of the five selected differentially expressed proteins was performed using IHC and WB in the additional 23 samples to confirm the results of proteomic analysis.

**Fig 6:**
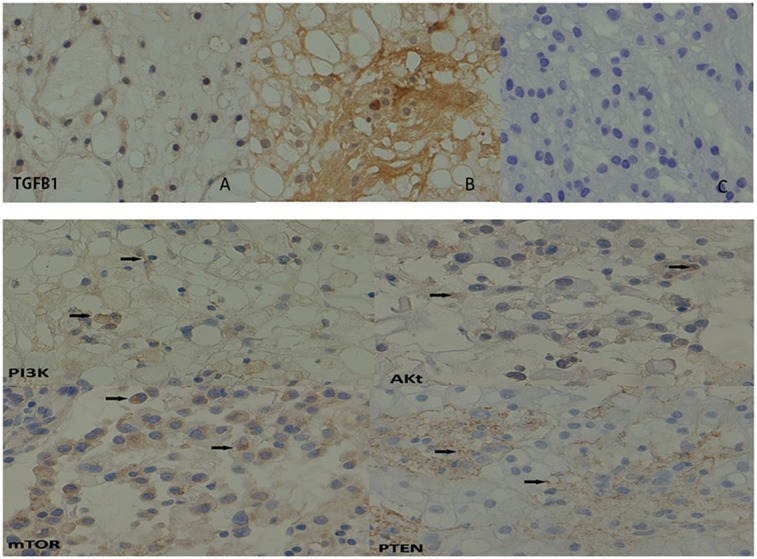
Results of immunohistochemical analysis ofTGFβ1, PI3K, Akt,mTOR and PTEN in tissue samples. Magnification: 200X. Representative images of clivus chordomas tissue that were immunostained for TGFβ1, PI3K, Akt, mTOR and PTEN. Graph a shows the representative positive image of TGFβ1 in exophytic type. Graph b shows the representative positive image of TGFβ1 in endophytic type. Graph c is a negative control.

In the study of IHC, the TGFβ1 protein in positive cells are located intracellularly, and the positive expression rate in the confirmation group was 95.6% (22/23). Compared with its expression in endophytic clivus chordoma (Type I), TGFβ1 expression in exophytic clivus chordoma (Type II) was greater; the difference between the two values was significant (*p* = 0.033), as shown in Fig. 6, which is consistent with the results from the differential proteomic analysis. The mTOR protein is located in the cytoplasm of chordoma cell. The positive expression rate in the confirmation group was 80.7% (20/23); there was no significant difference (*p* = 0.092) in the expression of mTOR between Type I and II, which is also consistent with the results from the differential proteomic analysis. PTEN expression in the confirmation group was 16/23 (69.6%). Expression of the endophytic type (Type I) PTEN was significantly lower than that of the exophytictype, and the differences between the two types were significant (*p* = 0.004). There was no significant difference in the expression of PI3K (*p* = 0.125) and Akt (*p* = 0.254) between Type I and II. The results are shown in Table 4.

**Table 4:** Immunohistochemical staining results of TGFβ,mTOR, and PTEN.

proteins	staining intensity	exophytic type (Ⅱ)	endophytic type (Ⅰ)	P value
TGFβ1	0	1	0	0.033
	1	0	3	
	2	7	7	
	3	5	0	
mTOR	0	0	1	0.092
	1	1	0	
	2	5	8	
	3	7	1	
PTEN	0	0	7	0.004
	1	4	1	
	2	7	2	
	3	2	0	

**Fig 7:**
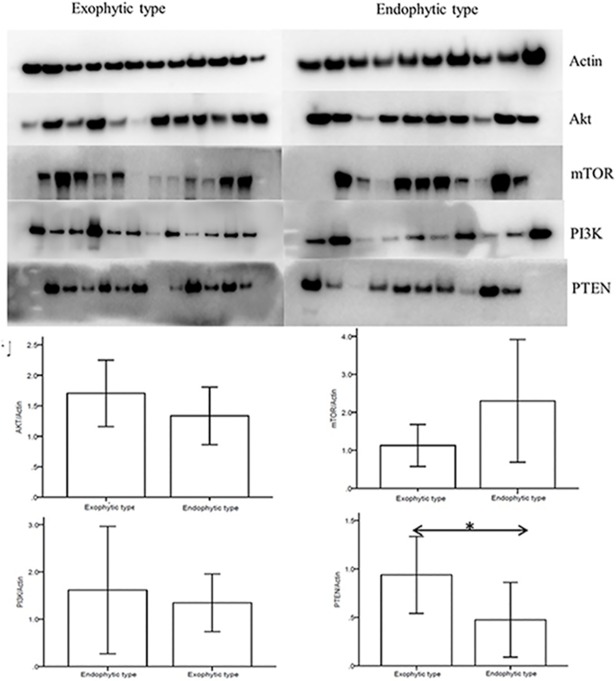
Western blot analysis for PI3K, Akt, mTOR and PTEN in 23 additional tissue samples. Graph a shows that high levels of PI3K, Akt, mTOR were detected in both exophytic and endophytic clivus chordomas. In contrast, the expression levels of PTEN in both exophytic and endophytic clivus chordomas were relatively lower. Graph b shows the quantification of expression levels using densitometry. The mean values of each group are represented in the bar graph; * p<0.05.87x81mm (600 x 600 DPI).

Furthermore, these four candidate proteins (PI3K, Akt, mTOR and PTEN) were validated using WB of the same additional 23 samples, and a quantitative analysis of the results was performed (Fig. 7). As shown in the graph, statistically significant differences were found in PTEN between endophytic type and exophytic type, which is consistent with the results from the differential proteomic analysis and IHC. The expression of mTOR level was higher in endophytic type and the level was higher in exophytic type, but there were no spastically significant differences for these two proteins. There were not any difference for PI3K level between endophytic type and the exophytic type.

## Discussion

Significant differences in the extent of skull base chordoma bone invasion exist, and they directly affect the extent of surgical resection that is possible[2]. For clivus chordoma with extensive infiltration of the skull base bone, surgical resection is difficult. In contrast, surgery more easily achieves subtotal or total resection of lesions with minor bone destruction. Based on the positional relationship between the tumor and bone clivus, this study classifies tumors as endophytictype (Type I)or exophytictype (Type II) to distinguish the degree of tumor invasion into the clivus bone. Endophytic tumors are typically within the clivus bone, and show expansive growth in the clivus withsevere destruction in the clivus bone. In contrast, thoughthe exophytic tumor is located at the clivus, the majority of it lies outside the clivus bone region, and the clivus bone destruction is less prominent. The classification of these two types of tumor can be used for research on the bone invasiveness of clivus chordoma, and the clinical prognosis of this two growth patterns was different, which will not be discussed in this paper.

This study is the first application of iTRAQ-based quantitative differential proteomic methods in determining the extent of bone invasion of clivus chordoma. By this high-throughput proteomic quantification method, 2251 proteins were quantified and 250 differntial proteins were discovered. According to the GO and IPA analysis of differential proteins, we discovered that the inflammatory cells especially phagocytic cellsin endophytic chordoma tissues,, exhibited significantly increased motor function; meanwhile, the expression levels of extracellular matrix proteins and cytoskeletal proteins generally decreased. It is speculated that increased inflammation and decreased expression of cytoskeletal proteins played a facilitating role in bone invasion of chordoma.

This study confirmed that the expression level of the upstream regulatory molecule TGFβ1 was significantly lower in the endophytic type than in the exophyticones. TGFβ1 is widespread in the human leukocyte antigen system, and it can inhibit the inflammatory response; it can also play an important role in tumorigenesis by promoting tumor metastasis and angiogenesis, as well as changing the microenvironment and evading immune attack [11]. Accordingly, we hypothesized that due to the low level of TGFβ1 expression, the skull base chordoma on one hand promotes inflammation by negative feedback. On the other hand, it directly causes the downregulation of downstream extracellular matrix proteins and cytoskeletal proteins, thereby promoting the invasion and destruction of bone. Therefore, we imply that TGFβ1 plays an important role in skull base chordoma bone invasion.

PTEN, as a tumor suppressor gene, regulate multiple signal transduction pathways that function in cell growth, cell migration and apoptosis [12]. By immunohistochemical staining and Western blot analysis in this study, it was discovered that PTEN levels were lower in endophytic chordoma than in exophytic chordoma. Therefore, PTEN expression levels may be associated with the degree of bone invasion by chordoma and with tumor texture. PTEN is a known negative regulator of PI3K proteins, and it can inhibit PI3K-Akt-mTOR signaling [13–15]. Low expression of PTEN could lead to the upregulation of mTOR, which is related to the poor clinical prognosis of sacral chordoma[13].

Both TGF β1 and PTEN regulate cell proliferation through a variety of signaling pathways, including mTOR signaling pathway[16,17]. The mTOR signaling channel has been regarded as an important channel for intracellular signal transduction that affects cell growth, tumor formation and cell invasion, including chordoma[18,19]. However, whether the mTOR signaling pathway is regulated by TGFβ1 to participate in bone invasion has not been reported. In this iTRAQ proteomic research, the mTOR signaling pathway has not been proved to be related with bone invasion as demonstrated in Fig. 4A. Moreover, IHC staining and Western blot confirmed that although the mTOR expression in chordoma cells was relatively high, there was no statically significant difference in the expression levels of mTOR, PI3K, and AKt in different subtypes of clivus chordomas. The activation of the PI3K-Akt-mTOR might mainly through the phosphorylation status but not through the expression levels. Thus, that the role of PI3K-AKt-mTOR signalingin the pathological process of bone invasion by clivus chordoma and the mechanism of the PI3K-AKt-mTOR signaling in clivus chordoma needs to be further studied.

Several publications reported that the TGF-β1 could activate the PI3K/Akt/ mTOR pathway, and further activate the downstream proteins, p70S6K. Thus, the TGF-β1 and PTEN are two important tumor related proteins which all regulated PI3K/Akt/ mTOR pathways. However, the mTOR pathway was partially activated in endophytic chordoma than the exophytic chordoma, but the TGF-β1 was down regulated in endophytic chordoma. The role of TGF-β1 in PI3K/Akt/mTOR pathways in chordoma needs further studied.

This study is methodologically innovative, but there are still some limitations, including a limited number of patients, which limited the application of statistical methods. Although differential proteomics research methods can reveal a large number of differentially expressed proteins, due to the limitation of validation method, only a small amount of protein could be verified, the high-flux MRM will be used for verification in the future. The analysis method depended on using currently known protein functions, and therefore, useful information was likely overlooked.

## Conclusion

Depending on the extent of bone invasion by clivus chordoma, the tumors can be divided intoendophytic and exophytic types by imagings. By integrating proteomic, IHC and Western blot’results, it was found that TGFβ1 may play an important role in bone invasion by clivus chordoma. The mechanisms may be related to mediating an increased inflammatory cell response and a decline in cytoskeletal protein expression. The expression level of PTEN may be associated with the degree of bone invasion by chordoma tumor. The exact signaling pathway through which TGFβ1 and PTEN play a role in clivus chordoma bone invasion remains to be confirmed by further studies.
